# From Global to Local: Semantic-Aware Instance-Wise Feature Selection

**DOI:** 10.3390/e28070809

**Published:** 2026-07-16

**Authors:** Zihan Wang, Yue Zhang, Hengpeng Xu, Zhenglu Yang, Jun Wang

**Affiliations:** 1 College of Mathematics and Statistics Science, Ludong University, Yantai 264000, China; wangxunxiaoju@163.com; 2 College of General Education, Yantai City College of Science and Technology, Yantai 264000, China; zy061322@163.com; 3 Tianjin Key Laboratory of Wireless Mobile Communications and Power Transmission, College of Electronic and Communication Engineering, Tianjin Normal University, Tianjin 300387, China; xuhp@tjnu.edu.cn; 4 Tianjin Key Laboratory of Network and Data Security Technology, College of Computer Science, Nankai University, Tianjin 300350, China; yangzl@nankai.edu.cn; 5 Key Laboratory of DISSec, Ministry of Education, Tianjin 300350, China

**Keywords:** feature selection, semantic correlation, inconsistent instance, multi-granularity learning

## Abstract

Feature selection is a promising dimension reduction technology that focuses on a reduced subspace by selecting excellent features. Most existing approaches tend to emphasize the discriminative ability of features based on either a global or a local evaluation criterion alone, and a few holistic approaches explore their selection granularity beyond the instance level. This study presents a novel Semantic-aware Instance-wise Feature selection model, dubbed SIF, to address the weakness of existing methods, which assess the significance of features from an individual view. Furthermore, SIF proposes to specify feature representations at the instance level, which is rarely touched by existing methods given the considerable learning complexity. In particular, SIF is designed as a sequential pipeline framework. First, it explicitly models semantic correlations and employs this information to select semantic-aware features. Then, inconsistent instances are captured and guide the instance-wise feature selection. Both types of features constitute the final optimal feature subset, which can represent semantics at a global level as well as describe instance characteristics at a local level. An extensive experimental evaluation illustrates the superiority of SIF under various metrics.

## 1. Introduction

With the data explosion, high dimensionality is becoming increasingly prominent, especially in machine learning and data mining fields [1]. Feature selection holds a pivotal place in reducing dimensions, which can provide a discriminative low-dimensional subspace for downstream tasks [2], not only decreasing their learning consumption but also boosting their learning performance [3]. In contrast to feature extraction, feature selection can preserve the semantics of original data. This advantage makes the subspace constructed by a feature selection model excel in its interpretability, which is vital for protein analysis, medical diagnosis, text categorization, and image classification [4].

**Figure 1:**
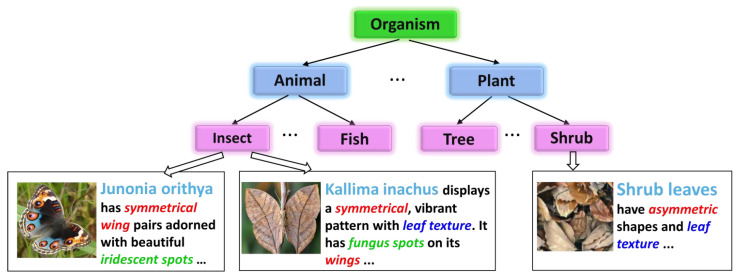
An example of semantic-aware and instance-wise representations.

As mentioned above, semantics is the key information to drive feature selection. The mainstream strategy of existing methods is to treat semantics in a flat manner. For instance, EALasso [5] and MLReliefF [6] deem semantic labels as independent classes and extend traditional lasso and ReliefF models for multi-label feature selection, respectively. LLSF [7] embeds semantic labels as a whole into a linear regression model for an optimal feature subset. In terms of the benefits of this strategy, flat semantics provides a straightforward as well as effective way to determine the global representation capability of features. However, semantic correlations are pervasive in reality, which can serve as a great help for feature selection. As illustrated in Figure 1, biological semantics naturally forms a hierarchical structure and shows inherent correlations. Given the close semantic correlation, the features that can better represent Kallima inachus are probably better at describing Junonia orithya. For instance, we can employ the features “symmetrical” and “wings” to distinguish these butterflies from fish, trees, and shrubs. These features play a vital role, especially when distinguishing highly similar objects, such as Kallima inachus and shrub leaves, both of which exhibit leaf textures.

A branch of existing methods, hierarchical feature selection, has been emerging based on modeling semantic hierarchy [8]. For instance, HFSGR [9] enforces relational graph constraints between different semantic granularities for feature selection. HFSDK [10] selects features for different semantic levels and eliminates data anomalies through upper-bound hinge loss. HiRRfam-FS [11] takes into account the semantic correlation in its selection process based on recursive regularisation. In general, these approaches select features for different-level semantics. However, they rarely propose to explicitly model semantic correlations and employ this information to guide feature selection. Furthermore, how to select features at the instance level has yet to be solved. In Figure 1, discriminating Kallima inachus from other butterflies calls for help from fine-grained representations due to their shared semantic features. Instance-wise features, such as “fungus spots”, are crucial in this context, as most butterflies exhibit iridescent spots.

To address these issues, we propose SIF in this study, a Semantic-aware Instance-wise Feature selection model. SIF selects semantic-aware and instance-wise features in a two-stage framework. That is, semantic-aware features are determined by the feature weight matrix constructed based on the semantic correlation matrix and feature-semantics affinity matrix. Then, inconsistent instances are positioned by the inconsistency matrix and SIF tailors fine-grained features for this kind of instances. The semantic-aware and instance-wise features are combined to form the final optimal feature subset, which represents different-level rich semantic knowledge for global recognition performance as well as specified characteristics at the instance level.

The main contributions are summarized as follows:

- A semantic correlation modeling mechanism is proposed to explicitly quantify semantic relationships among arbitrary label pairs and guide semantic-aware feature selection from a global perspective.
- A semantic inconsistency analysis strategy is developed to identify instances whose characteristics are insufficiently represented by common semantic features, enabling an instance-wise feature representation at a fine-grained level.
- An extensive experimental comparison is conducted on the openly available benchmark between SIF and ten comparison models using three multi-label classifiers, and SIF proves its competitiveness in diverse scenarios.

The other sections are organized as follows: Section 2 discusses the previous main research work and leads to the studies of this paper; Section 3 introduces the framework of SIF and gives its algorithmic flow; Section 4 describes the experimental configurations and experimental analyses in diverse scenarios; Section 5 provides an in-depth discussion of certain technical details and application considerations for SIF; Section 6 analyzes the potential limitations of SIF in detail; and Section 7 concludes the paper.

## 2. Related Work

Feature selection makes great sense for learning high-dimensional and complex data due to its effectiveness in reducing dimensions [12]. Feature selection methods are capable of decreasing computation time, enhancing predictive performance, and better understanding data in machine learning and pattern recognition applications. Based on the availability of labeled data, feature selection approaches can be categorized into supervised, unsupervised, and semi-supervised ones [13]. Supervised feature selection approaches take the majority in this family due to thei promising performance under the guidance of abundant labeled information [14]. This study lies in this line. Table 1 summarizes the selection mechanisms of the existing methods introduced in this section.

A critical role in directing the development of supervised feature selection is semantics [15]. In addition to EALasso [5] and MLReliefF [6] that have been introduced above, other representative methods show potential in modeling semantics. MCFS [14] selects discriminative features based on trace ratio in a richer subspace rather than being limited with the number of classes. ARMLNRS [16] evaluates by recognitive ability of features by modeling feature dependency and neighborhood proximity based on rough sets. BSFS [17] selects both discriminative features and those that can reveal the balanced structure of data on the basis of spectral clustering analysis. CD-LSR [18] introduces the $l_{2,0}$-norm with the simple least squares regression for feature selection. DHLI [19] provides a splitting semantic structure to avoid noise and preserve complementary information in feature selection. MSFS [20] breaks down multi-label semantic information to capture semantic concepts between multiple labels. (AF)^2^-S3Net [21] combines the semantic information of the environment and uses a unique adaptive feature selection module to achieve feature mapping reweighting. Generally speaking, the above approaches learn semantics in a flat manner, without delicate consideration of semantic correlations. In contrast, this study introduces a novel semantic-aware feature selection model which employs semantic correlations to find different kinds of features. Semantic correlations are embedded in each selection process and help find discriminative features at both semantic and instance levels.

With the growing emphasis on semantic analysis, semantic-aware feature selection methods have garnered extensive research attention in recent years, emerging as a critical paradigm for dimension reduction. LLSF [7] learns second-order and high-order semantic correlations to guide feature selection. SDIM [22] maximizes the latent semantic differences of two instances based on semantic dissimilarities to deal with label ambiguities. OM-LEFSD [23] utilizes the latent semantic information in each instance to supervise online streaming feature selection. ACGA-RNB [24] explores a semantic sampling method to perform wrapper-based similarity feature selection. FSSS [25] calculates similarity scores between labels as semantic regularisation to steer feature selection. These semantic-aware methods perform feature selection by mining diverse semantic relevance, yet they merely evaluate features at the coarse semantic level. This strategy overly emphasizes features’ global discriminative capabilities while neglecting instances’ local recognition details. In contrast, SIF explores instance-wise features and constructs a multi-granularity feature selection framework, enabling a comprehensive representation of the learning target.

**Table 1:** A summary of the representative feature selection methods.

Category	Methods	Explicit Semantic Correlation	Multi-Granularity	Instance-Level Representation
Traditional Multi-Label	EALasso [5]	×	×	×
	MLReliefF [6]	×	×	×
	MCFS [14]	×	×	×
	ARMLNRS [16]	×	×	×
	BSFS [17]	×	×	×
	CD-LSR [18]	×	×	×
	DHLI [19]	×	×	×
	MSFS [20]	×	×	×
	AF^2^-S3Net [21]	×	×	×
Semantic-Aware	LLSF [7]	√	×	×
	SDIM [22]	√	×	×
	OM-LEFSD [23]	√	×	×
	ACGA-RNB [24]	√	×	×
	FSSS [25]	√	×	×
Hierarchy-Guided	HFSGR [9]	×	√	×
	HFSDK [10]	×	√	×
	HiRRfam-FS [11]	×	√	×
	HCFS [26]	×	√	×
	HFSLM [27]	×	√	×
	HFSLE [28]	×	√	×
Proposed Method	SIF	√	√	√

In the family of semantic-aware feature selection methods, one emerging branch focuses on capturing excellent features at diverse granularities, known as hierarchical feature selection [29]. In particular, the corresponding learning task can be deemed as hierarchical classification, where the knowledge of hierarchical structures is used to select an optimal feature subset [30]. HFSGR [9], HFSDK [10], and HiRRfam-FS [11] that have been mentioned before are located on this track. In terms of other excellent approaches, HCFS [26] employs a multi-granularity clustering structure to model semantic hierarchy. HFSLM [27] leverages a large-margin nearest-neighbor strategy built in the hierarchical classification scenario to accomplish the distance metric-based feature selection. HFSLE [28] integrates the frameworks for hierarchical label enhancement and hierarchical feature selection based on the label distribution paradigm. Most hierarchical feature selection methods evaluate semantic relationships through tree structures, limiting their scope to parent-child and sibling dependencies. In other words, they cannot explicitly model semantic correlations from a global view. Furthermore, the aforementioned approaches rarely discuss how to explicitly evaluate semantic correlations as well as how to specify features for instances. Compared to these approaches, SIF quantifies semantic correlations between arbitrary pairwise semantic labels, which is more comprehensive and can capture the global discrimination information in semantic space. Furthermore, SIF can model instance-wise representations to achieve a more fine-grained target description.

In this paper, the semantic-aware feature selection model SIF is constructed. In the framework of SIF, semantic-aware feature selection is performed to facilitate semantics representation, and then, instance-wise feature selection is implemented to describe recognition details. Different granular features are comprehensively selected to portray general and detailed information at diverse levels.

## 3. SIF

**Figure 2:**
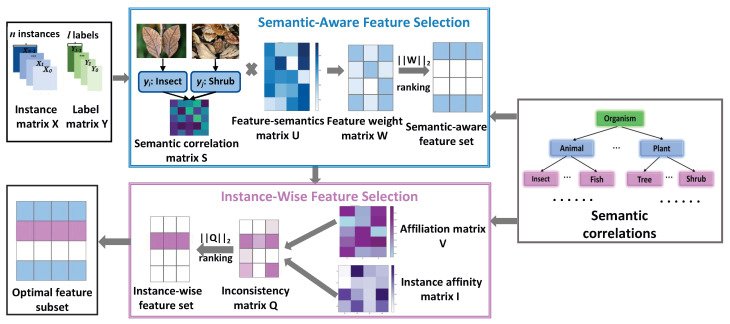
The diagram of the SIF model.

This section introduces the novel semantic-aware feature selection model specifying, for instances, SIF. As shown in Figure 2, SIF selects semantic-aware features at the first stage based on semantic correlations and finds instance-wise features at the second stage. Eventually, different-granular features are refined to build the final set.

### 3.1. Semantic-Aware Feature Selection

In reality, there exists complex correlation and hierarchical relationship in semantics. We tend to find the features that can globally describe the commonly shared general semantics in the semantic-aware feature selection. For example, Junonia orithya and Kallima inachus have common features “symmetrical” and “wings”, and selecting this kind of features helps discriminate these butterflies from fish, trees, and shrubs, as shown in Figure 1. Suppose $X=\left[x_{1};\ldots ;x_{n}\right]\in R^{n\times d}$ represents the instance matrix where each instance is characterized by *d* features $F={\left\{f_{j}\right\}}_{j=1}^{d}$ and annotated by the semantic label set $Y={\left\{y_{c}\right\}}_{c=1}^{l}$, where $y_{c}={\left\{0,1\right\}}^{n}$. We resort to the Hilbert–Schmidt Independence Criterion (HSIC) [31] to model the semantic correlation that can dispense with data discretization or distribution estimation [32]. Specifically, we determine the correlation between the semantic labels $y_{i}$ and $y_{j}$ based on their HSIC score as follows:

(1) $$\mathrm{HSIC}\left(y_{i},y_{j}\right)={\left(n-1\right)}^{-2}\mathrm{tr}\left(H\phi \left(y_{i}\right)H\phi \left(y_{j}\right)\right),\;\phi \left(y_{c}\right)=E\left[k\left(y_{c},y_{c}^{T}\right)\right],\;c=i,j,$$

where $H=y_{i}y_{j}^{T}-\left(1/n\right)ee^{T}\in R^{n\times n}$, e is an all-one column vector, and tr(·) represents the trace of the matrix. $\phi (y_{c})$ is defined as the expectation over the semantic label $y_{c}$ based on the kernel *k*. Here, we employ the linear kernel and compute $k\left(y_{c},y_{c}^{T}\right)$ as $y_{c}y_{c}^{T}$.

HSIC [33] can effectively capture linear and nonlinear dependencies through kernel embedding and does not depend on explicit assumptions of the data. In the calculation process, HSIC uses a simple empirical formula of the kernel matrix, which is numerically stable in the face of large-scale multi-label data. Compared with mutual information, which is an effective and theoretically sound evaluation metric, HSIC naturally supports dependency measurement for variables of arbitrary distributions, dispensing with any probability estimation. In contrast to another widely used dependency measurement, distance correlation, HSIC offers greater flexibility rather than being restricted to Euclidean distance and can be applied to variables of different data types by defining appropriate kernels.

In Equation (1), the kernel $k(y_{c},y_{c}^{T})$ maps the semantic label $y_{c}$ to the Reproducing Kernel Hilbert Space (RKHS), facilitating the estimation of non-linear dependencies of $y_{c}$ with other labels. The expectation $E\left[k(y_{c},y_{c}^{T})\right]$ over the label distribution is calculated to alleviate the influence of random noise. The term $H\phi \left(y_{i}\right)H\phi \left(y_{j}\right)$ quantifies the independence of $y_{i}$ and $y_{j}$ in the RKHS, where H is a centering matrix. This matrix ensures the kernel matrices are properly centered, thereby isolating the intrinsic statistical relationship between variables by removing mean-induced biases.

Then, we can obtain the semantic correlation matrix S as follows:

(2) $$S={\left[s_{ij}\right]}_{i,j=1}^{l}\in R^{l\times l},s_{ij}=HSIC\left(y_{i},y_{j}\right).$$

A higher $s_{ij}$ indicates a closer correlation between the two semantic labels $y_{i}$ and $y_{j}$, such as Junonia orithya and Kallima inachus. Accordingly, a feature is probably discriminative for $y_{i}$ if it is discriminative for $y_{j}$. It is worth noting that the semantic correlation matrix S depends on the estimation of HSIC, which measures the dependence between two random variables by computing the Hilbert–Schmidt norm of their cross-covariance operator in a reproducing kernel Hilbert space. This metric remains non-negative, with a value of zero indicating variables’ independence, and it is invariant to variables’ directions. In other words, it does not produce negative values when the physical directions of the variables are reversed. Accordingly, all elements in the matrix S are non-negative.

Similarly, we can readily induce the feature-semantics matrix U based on HSIC as follows:

(3) $$U={\left[u_{ij}\right]}_{i=1,\ldots ,d,j=1,\ldots ,l}\in R^{d\times l},u_{ij}=HSIC\left(f_{i},y_{j}\right).$$

The feature $f_{i}$ is related to a higher $u_{ij}$ if it can better represent the semantic label $y_{j}$.

Based on S and U, we can finally determine the feature weight matrix as

(4) $$W=U\times S\in R^{d\times c},$$

which describes the representation capability of each feature at the semantic level. We can rank features according to their general semantic scores by calculating the $\ell_{2}$-norm of W in rows (i.e., $\left|\right|W^{i}{\left|\right|}_{2},i=1,\ldots ,d$), and select top-$k_{1}$ features with the highest $\left|\right|W^{i}{\left|\right|}_{2}$ values to form the semantic-aware feature subset.

### 3.2. Instance-Wise Feature Selection

In the face of complex and tremendous data, it is hard to yield satisfactory learning performance merely via semantic-aware features. This is mainly due to the fact that excessively focusing on the high-level representation information may ignore detailed and specific information. In this section, instance-wise features are further selected based on the semantic-aware feature subset obtained in Section 3.1.

**Figure 3:**
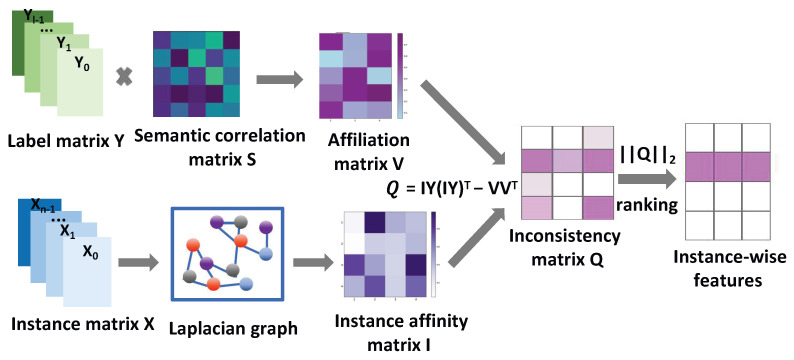
The process of the instance-wise feature selection.

An ideal solution is to examine the distinctive characteristics of each instance and formulate a feature selection matrix for them. Considering the huge complexity of this solution, however, we resort to a compromised strategy; that is, we capture inconsistent instances and specify features for this kind of instances. Inconsistent instances show specific recognition details in contrast to the other instances with the same semantic label. For instance, Kallima inachus in Figure 1 exhibits unique morphological traits that are absent in other butterflies, such as its “leaf texture” and “fungus spots”. The identification and selection of these kind of features are critical for accurate species recognition. In other words, semantic-aware features portray the recognition commonality of a majority of instances, such as “symmetrical”, “wings”, and “slender antennas” for butterflies. In contrast, instance-wise features delineate the recognition diversity of a minority of instances, such as Kallima inachus’s specialized patterns. The instance-wise feature selection process is delineated in Figure 3. We identify inconsistent instances by quantifying their semantic deviation in the feature space. Specifically, an instance will be annotated as an inconsistent one if its semantics are disparate from those of its tightly correlated instances. First, we define the affiliation matrix of instances w.r.t. semantic labels as

(5) $$V=YS\in R^{n\times l},$$

where Y is the semantic label matrix and S is the semantic correlation matrix obtained in Equation (2). The matrix V describes the belonging degree of instances to semantic labels under the control of semantic correlations, and a high $V_{ij}$ indicates that the *i*-th instance tends to bear the *j*-th semantic label.

Then, we construct the instance affinity matrix via the Laplacian graph as

(6) $$I={\left[I_{ij}\right]}_{i,j=1}^{n}\in R^{n\times n},I_{ij}=\left\{\begin{array}{c} \alpha_{ij},\;y_{i}=y_{j}\\ 0,\;otherwise \end{array}\right.,$$

where $\alpha_{ij}$ is the affinity degree between the instance $x_{i}$ and the instance $x_{j}$, which is defined as $\alpha_{ij}=\exp\left(-{∥x_{i}-x_{j}∥}^{2}/2\delta^{2}\right)$. $\delta^{2}$ is the adjustable parameter and fixed as $\delta^{2}=mean\left({∥x_{i}-x_{j}∥}^{2}\right)$ in this study. The Gaussian kernel (a.k.a. Radial Basis Function) is a widely adopted kernel function in feature selection due to its ability to capture nonlinear dependencies between variables [34,35]. In contrast to other kernels, such as linear or polynomial kernel functions, the Gaussian kernel’s universal approximation property and locality sensitivity make it particularly effective for identifying complex, non-monotonic relationships in high-dimensional data. Given the complex multi-label correlation of instances, this study adopts the Gaussian kernel to determine the instance affinity degree.

The matrix I describes the similarity of instances in the original feature space, and the instances close to each other in this space should share a tight semantic correlation in the reduced feature space. Based on this assumption, we construct the following inconsistency matrix Q to explore the potentially inconsistent instances:

(7) $$Q=IY{\left(IY\right)}^{T}-VV^{T},$$

Given I reflecting the neighborhood relationship between instances in the original feature space, IY reflects the semantic information induced by adjacent instances. Correspondingly, $IY{\left(IY\right)}^{T}$ can be deemed as the semantic similarity of pairwise instances observed in the feature space. On the other hand, we use Equation (5) to calculate the label correlations, i.e., V. Accordingly, the matrix $VV^{T}$ represents the semantic similarity observed in the label space. As a result, their deviation, i.e., the inconsistency matrix Q can be leveraged to the measure the semantic difference of adjacent instances in the dual spaces. Specifically, $Q^{i}$ denotes the semantic difference of the instance $x_{i}$ from its adjacent instances, where $Q^{i}$ is the *i*-th row of the matrix Q. A larger $\left|\right|Q^{i}{\left|\right|}_{2}$ value indicates a more significant semantic difference of $x_{i}$, and vice versa. In other words, $\left|\right|Q^{i}{\left|\right|}_{2}$ portrays the semantic inconsistency of $x_{i}$.

**Figure 4:**
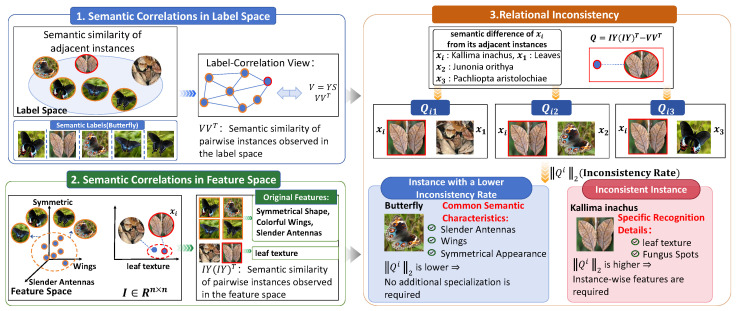
An intuitive illustration of the inconsistent instance identification.

We further provide an intuitive illustration concerning the concept of instance inconsistency in Figure 4. In the label space, the adjacent structure of butterflies is constructed via the affiliation matrix V, and $VV^{T}$ is further built to explore their potential global semantic similarity observed in the label space. In the feature space, the instance affinity matrix I can capture their local neighborhood relationships, and $IY{\left(IY\right)}^{T}$ represents the semantic similarity observed by the original features, such as “symmetrical shape”, “colorful wings”, and “slender antennas”. Their deviation, the inconsistency matrix Q, can quantify semantic-feature inconsistency of each instance in the dual spaces. For example, the inconsistency score of Junonia orithya is lower, indicating that it can be well described by the common semantic features. In contrast, the inconsistency score of Kallima inachus is higher, requiring more specific recognition representations in detail.

We can calculate the $\left|\right|Q^{i}{\left|\right|}_{2}$ value for each instance, which represents the inconsistency rate of the *i*-th instance. Based on these inconsistency rates, we can select θ percent of the instances with the highest inconsistency rates as the inconsistent ones. The effects of θ will be evaluated in Section 4.3.4.

In the previous stage, we have built the semantic-aware feature subset to represent the learning target at a coarse-grained level. However, the representation requirements of inconsistent instances are distinguished from the semantically consistent ones. Consequently, we adopt conditional mutual information (CMI) [36] as the feature evaluation criterion as follows:

(8) $$Score\left(f_{j}\right)=\sum_{f_{i}\in F_{c}}\sum_{c=1}^{l}MI\left(f_{j},y_{c}|f_{i}\right),$$

where $F_{c}=\left\{f_{1},f_{2},\ldots ,f_{k}\right\}$ represents the semantic-aware feature subset built in the first stage, $f_{j}\notin F_{c}$ is the candidate feature, and $MI\left(f_{j},y_{c}|f_{i}\right)$ is the mutual information of $f_{j}$ and $y_{c}$ given $f_{i}$ as the condition.

The main reason for using the CMI criterion lies in its advantages in the following aspects: (1) addressing the issue of feature complementarity between the semantic-aware features and instance-wise features; (2) eliminating the dependency on any off-the-shelf learning models while remaining non-sensitive to model parameter configurations; and (3) providing a strong theoretical grounding and solid empirical performance, which have been widely validated in existing feature selection methods [37,38].

Then, we rank all candidate features by their scores and select the top-$k_{2}$ highest-scoring features as the instance-wise features. These fine-grained features are discriminative for the inconsistent instances and helpful to express the specific characteristics of the learning target. The instance-wise feature subset is combined with the semantic-aware feature subset obtained in Section 3.1 to formulate the final optimal subset.

### 3.3. The Framework of SIF

The whole selection process of SIF is summarized in Algorithm 1, which is divided into two stages. In specific, steps 2 to 5 form the process of selecting semantic-aware features and steps 6 to 13 correspond to the process of selecting instance-wise features. The effects of the inconsistency rate θ and the number of two kinds of features $k_{1}$ and $k_{2}$ will be evaluated in Section 4. In the process of selecting instance-wise features, the top θ% instances are selected to construct the inconsistent instance set. In the SIF framework, inconsistent instances are identified after the semantic-aware feature selection stage and remain fixed throughout the second stage of instance-wise feature selection. A major reason for adopting a fixed configuration is that iteratively updating inconsistent instances would inevitably incur additional computational cost, imposing a high burden on SIF.
**Algorithm 1** SIF**Input:** X, Y, θ, $k_{1}$, $k_{2}$**Output:** the optimal feature set $F^{*}$  1:**begin**  2:construct the semantic correlation matrix S via Equation (2);  3:construct the feature-semantics matrix U via Equation (3);  4:compute the feature weight matrix W=S×U via Equation (4);  5:select top-$k_{1}$ features with the highest ${\left||W|\right|}_{2}$ scores to construct the semantic-aware feature subset $F_{c}$;  6:construct the affiliation matrix V according to Equation (5);  7:construct the instance affinity matrix I via Equation (6);  8:calculate the inconsistency matrix Q via Equation (7);  9:select θ instances with the highest ${\left||Q|\right|}_{2}$ scores as the inconsistent ones;10:**for** each feature $f_{j}\notin F_{c}$ **do**11:      calculate $Score(f_{j})$ via Equation (8);12:**end for**;13:select top-$k_{2}$ features with the highest scores to build the instance-wise feature subset $F_{f}$;14:$F^{*}=F_{c}⋃F_{f}$;15:**end;**

The purpose of selecting semantic-aware features at the first stage is to better represent general semantics globally, and instance-wise feature selection is performed in the second part, on the basis of the subset of semantic-aware features, to distinguish the local recognition information in a detailed way. Finally, both semantic-aware and instance-wise features are simply combined for the final optimal feature subset. As to the complexity of SIF, the major consumption of selecting semantic-aware features is to construct S and U. When computing both matrices, the HSIC estimations regarding label-label and feature-label correlations are involved, where the consumption of constructing S and U takes $O(l^{4})$ and $O(d^{2}l^{2})$, respectively. Finding $k_{1}$ semantic-aware features approximately takes $O(k_{1}\log d)$ time. Given $k_{1}<<d$, the time consumption for the first stage is approximately equal to $O(l^{4}+d^{2}l^{2})$. At the second stage, finding inconsistent instances is one involved term which is $O(n^{2}l^{2})$. The time consumption of computing candidate features’ scores based on CMI is close to O(dnl), given $k_{1}<<d$. Thus, the total consumption of SIF comprising two stages is approximately equal to $O(l^{4}+d^{2}l^{2}+n^{2}l^{2}+dnl)$, where *d*, *l*, and *n* represent the numbers of features, semantic labels, and instances, respectively.

## 4. Experimental Evaluations

In this section, we will evaluate the performance of the proposed SIF on openly available benchmarks. Furthermore, the variants of SIF as well as the influence of its parameters will also be examined in this section.

### 4.1. Datasets

We collect seven groups of multi-label datasets (https://mulan.sourceforge.net/datasets-mlc.html (accessed on 12 July 2026)) for evaluations, whose detailed descriptions are displayed in Table 2:

- Enron: an email dataset from Enron Corporation, which records a large number of email messages including email titles and body contents;
- Medical: a clinical free-text dataset that collects outpatient chest radiography and renal surgery records over a one-year period;
- Bibtex: a book and label dataset that contains the information about book titles, authors, publishers, chapters, and organisations;
- Delicious: a social bookmarking dataset containing the records of Internet users’ tags on web pages and bookmarks in their personal bookmarks;
- Wireless: a wireless network dataset recording hourly average responses from five metal oxide chemical sensor arrays in an air quality chemical multi-sensor device;
- Scene: a multi-label scene classification dataset categorizing images into semantic classes, including “beach”, “sunset”, “fall foliage”, “field”, “mountain”, and “urban”;
- Corel5k: an object recognition dataset for machine translation, in which each image has different number of segments and each segment is represented by 36 features.

All feature variables in the raw data were standardized to have zero mean and unit variance (i.e., converted to z-scores). Most benchmark datasets contain nominal features, except for wireless and scene, which are numeric. To avoid the complexity of continuous probability density estimation and to accelerate the computation of CMI, we discretized these two datasets into five bins using an equal-width strategy.

More sophisticated discretizers, such as kernel density estimation and bandwidth optimization, can provide more accurate dependency estimation than fixed-bin discretization. However, these approaches introduce additional hyperparameters and generally incur higher computational costs. Consequently, we employ a five-bin discretization strategy to balance estimation reliability and computational efficiency. Additionally, the choice of five bins is a practical trade-off between estimation accuracy and statistical robustness. When the number of bins is excessively large, the estimated conditional probabilities become sparse, particularly for inconsistent-instance subsets containing a limited number of instances. In contrast, an overly coarse discretization may result in information loss and reduced discriminative capability. Therefore, a moderate discretization level is preferred to ensure reliable probability estimation while preserving sufficient feature variability.

We adopt the mutual information estimation toolbox provided in the original mRMR [39] work, as it offers a well-established and reliable implementation for this task.

### 4.2. Comparison Methods and Configurations

In order to comprehensively evaluate the performance of SIF, we compare SIF with the “Origin” method, wherein all of the original features without any selection are tested, reflecting the intrinsic performance of the classification methods in the absence of feature selection. Furthermore, seven popular multi-label feature selection approaches are as follows:

- LLFS (Learning Label-Specific Features) [7]: A semantic-aware feature selection method using an optimized linear regression model for feature selection;
- EALasso (Extended Adaptive Lasso) [5]: Extending the traditional adaptive Lasso model by automatically learning the optimal regularisation parameters using a genetic algorithm;
- MLReliefF (Multi-Label ReliefF) [6]: Identifying feature weights by measuring class similarities via nearest neighbours in the same class and class dissimilarities via different-class samples;
- ARNRS (Attribute Reduction using Neighborhood Rough Sets) [16]: Evaluating the discriminative ability of features by updating the feature dependency and neighborhood proximity based on neighborhood rough sets;
- BSFS (Balanced Spectral Feature Selection) [17]: A spectral feature selection method with a balanced regularization term to integrate balanced spectral learning with feature selection;
- CD-LSR (Coordinate Descent method for Least Squares Regression) [18]: A parameter-free optimization framework based on the coordinate descent method using the vanilla least square regression for feature selection;
- MCFS (MultiCenter points and local structure learning for Feature Selection) [14]: A supervised feature selection via multi-center and local structure learning based on trace ratio criterion.

In addition to the above approaches, we also compare SIF with three groups of hierarchical feature selection models that consider semantic correlations, including HFSGR, HFSDK, and HiRRfam-FS:

- HFSGR (Hierarchical Feature Selection with Subtree-based Graph Regularization) [9]: Implementing hierarchical feature selection via graph regularisation and two-way dependence among different classes;
- HFSDK (Hierarchical Feature Selection method Driven by data and Knowledge) [10]: A hierarchical method that captures compact feature subsets by splitting the original semantic space, superior in its robustness to data outliers;
- HiRRfam (Family Relationship-based Hierarchical Feature Selection with Recursive Regularization) [11]: Selecting different feature subsets for each node using parent-children and sibling relationships for hierarchical regularization.

In the experiments, we divide the benchmark into training and testing datasets randomly. Each approach selects features to construct the reduced subspace where the ML-KNN classifier is trained and tested. The feature subspace with high ML-KNN classification performance can be regarded as a discriminative space, which is conducive to recognition tasks and facilitates downstream applications. The ML-KNN classifier is employed due to its extensively verified performance in feature selection [40,41]. To ensure a comprehensive evaluation and assess the generalization performance of the compared methods, we also test two additional multi-label classifiers, that is, LIFT [42] and BiLAS [43]. LIFT is a state-of-the-art multi-label classifier that discriminates instances through clustering analysis using label-specific features. BiLAS is a recently proposed classifier, which induces prediction with BiLabel-specific features. In the following experiments, the number of nearest neighbors for ML-KNN is set to 3 and the smoothing parameter is 0.5. In terms of LIFT, its clustering ratio, which controls the scales of positive and negative instances, is fixed to 0.1 and the kernel type of its SVM induction learner adopts the linear one.

We use grid search optimization to capture the optimal parameters for each approach. As to EALasso, the penalty parameter $\lambda \in \{1\times 10^{-4},1\times 10^{-3},1\times 10^{-2},1\times 10^{-1},1\}$. The number of nearest neighbors of MLReliefF is searched within the set {1,3,5,10,20}, and the neighborhood parameter of ARMLNRS is searched in {0.1,0.2,0.3,0.4,0.5}. In terms of BSFS, the clustering balance parameter $\gamma \in [1\times 10^{-3},1\times 10^{3}]$. The penalty parameters α, β, and γ of LLSF and three hierarchical feature selection approaches (i.e., HFSGR, HFSDK, and HiRRfam-FS) are searched in the set of $\left\{1\times 10^{-3},1\times 10^{-2},1\times 10^{-1},0.3,0.5,0.7,0.9\right\}$. The number of multi-center points of MCFS is searched in {1,3,5,7,9}. As to SIF, the ratio of selecting semantic-aware features and instance-wise features (i.e., $k_{1}/k_{2}$) is searched in the subset {1/9,2/8,3/7,4/6,5/5,6/4,7/3,8/2,9/1} and its effects will be further evaluated in Section 4.3.2. The inconsistency rate θ belongs to {0.1,0.2,0.3,0.4,0.5,0.6} and will be evaluated in Section 4.3.4. We adopt four measurements, that is, average precision, F-measure, recall, and Hamming loss, for an extensive evaluation.

### 4.3. Experimental Results

#### 4.3.1. Classification Performance

In order to compare the feature selection performance of SIF versus the state-of-the-art methods, we conduct them on the seven groups of datasets and select 30% features on each benchmark for ten iterations. All compared methods, including SIF, are filter-based feature selection approaches. That is, they evaluate all features according to a certain metric and select the top-scoring ones to form the optimal feature subset. This mechanism differs from wrapper-based feature selection approaches, which output a fixed-size feature subset. To ensure a fair comparison, we fixed the feature selection ratio for all methods, which is a common configuration in filter-based settings [41,44]. As to the choice of a 30% selection ratio, a smaller ratio (e.g., 10%) may lead to the loss of important discriminant information, while a substantially larger ratio may introduce redundant features and significantly increase time consumption. As a balanced trade-off, we select 30% of the original features for all methods in comparison. We record the averaged ML-KNN classification performance over 20 times achieved in the subspace constructed by each selected feature subset in Table 3 and Table 4.

To facilitate comparative analysis, the optimal and suboptimal results on each dataset are labelled in bold and underlined, respectively. The “↑” notation indicates that the higher performance is better, while the “↓” notation is contrary. As can be seen from the two tables, the performance of SIF is superior or comparable to the other feature selection approaches under the four evaluation metrics, which proves the effectiveness of SIF in dimension reduction. In terms of the feature selection approaches without modeling semantic correlations, EALasso and MLReliefF perform comparably well. Both approaches are extended from single-label prototypes, whose excellent performance on single-label cases provide support for their success in multi-label scenarios.

#### 4.3.2. Ablation Studies

- **Effects of Each Component**

**Figure 5:**
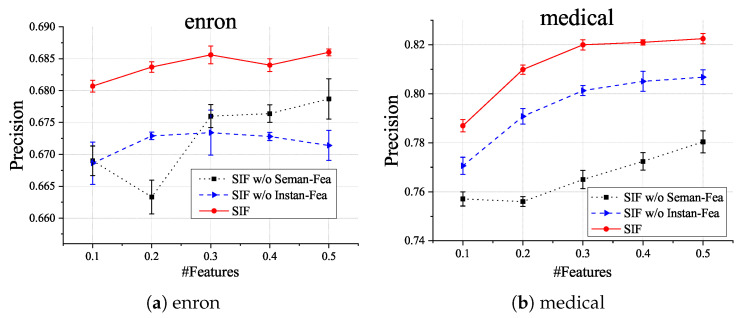
Effects of each component of SIF.

In order to evaluate the performance of each component of SIF, we evaluate its variants without semantic-aware features (SIF w/o Seman-Fea) and without instance-wise features (SIF w/o Instan-Fea) on the enron and medical datasets. We increase the number of selected features from 10% of the original features to 50% and demonstrate their average precision performance in Figure 5, where the error bar is depicted to reveal the performance variance over 20 times. As portrayed in the figure, SIF consistently takes priority over its variants without semantic-aware features or without instance-wise features, validating the significance of both feature selection components in dimension reduction. As to the optimal ratio for each dataset, enron is 0.7 and medical is 0.6.

We also observe that semantic-aware and instance-wise features might show disparate effects on different datasets. That is, the ML-KNN classifier seems to gain more benefits from the instance-wise features on enron, while it is more enhanced by the semantic-aware features on medical. This may be due to the distinctive characteristic of each dataset. The enron dataset collects the emails of the Enron Corporation covering finance, operations, and private communications. The topics of these emails are various, such as company, politics, trip, relationship, legal issues, and so on. The emotional tones of these emails are more diverse, including jubilation, humor, sadness, anger, anxiety, worry, concern, just to name a few. Therefore, these email instances show more diversity than commonality and specifying instance-wise features for them may be more favorable. In contrast, the categories of the medical dataset seem to be more centered, which are all ICD-9-CM labels from clinical texts. Correspondingly, selecting semantic-aware features to describe their coarse commonality is more beneficial.

Generally speaking, instance-wise features can better describe the instances with specific details, and semantic-aware features can represent common semantics and facilitate recognition of the instances with similar semantics. SIF captures multi-grained features and combines them to express the diversity and commonality inherent in complex semantic labels.

- **Effects of $k_{1}$ and $k_{2}$**

**Figure 6:**
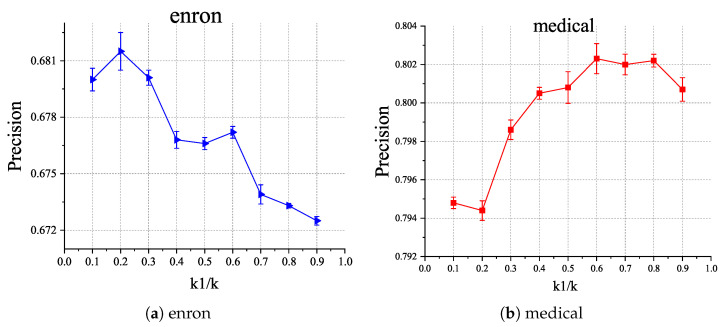
Effects of the scales of semantic-aware and instance-wise features.

We further evaluate each component by varying its portion in SIF, that is, the values of $k_{1}$ and $k_{2}$ which control the scales of selecting semantic-aware and instance-wise features. We fix the total number of selected features to 30% of the original ones and gradually increase the ratio of $k_{1}/k$. Figure 6 illustrates that a small scale of each kind of features may not be advantageous, which is consistent with the observation in Figure 5.

In contrast to Figure 5, which examines the superiority of different kinds of features in multi-label classification, Figure 6 focuses on the effects of these features on SIF. It is worth noting that there may be a consistent preference in the semantic-aware and instance-wise feature subsets. To elaborate, the semantic-aware feature subset is first constructed and then, the features excluded from this subset as well as those discriminative for inconsistent instances will be selected as instance-wise features. This suggests that certain features may be preferred by both subsets. For example, Kallima inachus has the common features as other butterflies, such as “symmetrical”, “wings”, and “slender antennas”. When merely specifying instance-wise features for Kallima inachus, the two features have a high potential of being selected. In addition to the characteristic of the enron data, this can explain the reason from another aspect why enron’s instance-wise feature subset performs well in Figure 5 and Figure 6. Despite the benefits of instance-wise or semantic-aware features, their individual performance remains inherently limited. SIF integrates their merits and achieves progress in selecting diverse features, which is proven effective in the two figures.

- **Effects of HSIC-Based Correlation**

The ablation experiment in the **Effects of Each Component** section validates the contribution of semantic-aware feature selection, yet it does not clearly indicate how much of the improvement stems from the HSIC-based correlation. To address this, we further compares SIF with its variant SIF-NC, SIF-DC(E), SIF-DC(M) and SIF-HD, which adopts a Native Correlation based on the Pearson correlation coefficient, Distance Correlation based on Euclidean distance and Mahalanobis distance, and Hoeffding’s D, respectively.

**Figure 7:**
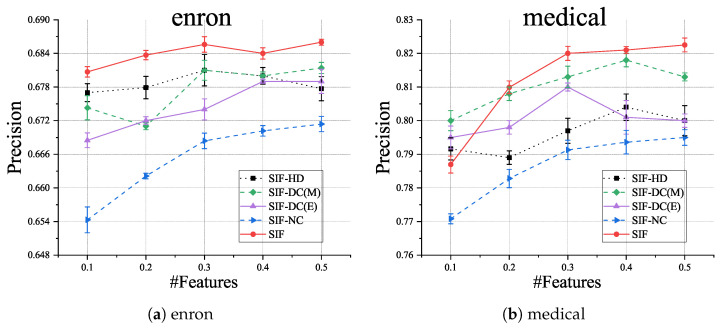
Comparisons of different correlation metrics.

As shown in Figure 7, SIF consistently outperforms SIF-NC, SIF-DC(E), SIF-DC(M), and SIF-HD on the enron and medical datasets. The Pearson correlation coefficient is a classical linear relationship measurement, which is effective in linear learning scenarios but loses its effectiveness in nonlinear ones. In contrast, HSIC can effectively capture the nonlinear relationships of random variants with arbitrary distributions and different data types. Although distance correlations and Hoeffding’s D are capable of capturing nonlinear relationships, HSIC offers superior flexibility and statistical power by detecting arbitrary non-linear dependencies through kernel functions, which facilitates its generalization to multivariate settings. Since the semantic relationships in multi-label datasets are typically complex and nonlinear, HSIC appears more appropriate for this learning task.

- **Effects of Inconsistency Detection Mechanism**

To further investigate the effectiveness of the proposed inconsistency detection mechanism and the CMI-based instance-wise feature selection strategy, we compare SIF with its variants, SIF-IF, SIF-KM, and SIF-mRMR. Specifically, SIF-IF and SIF-KM have the same configurations as SIF, except that they identify inconsistencies based on Isolation Forest [45] and K-means-based outlier detection [46], respectively. SIF-mRMR replaces the CMI-based strategy with the well-known mRMR criterion [47], which selects optimal features by maximizing feature-label relevance and minimizing feature-feature redundancy. In the experiment, the contamination of SIF-IF is equal to θ and fixed to 0.3, and the number of clusters for SIF-KM is equal to the number of labels, i.e., *l*.

**Figure 8:**
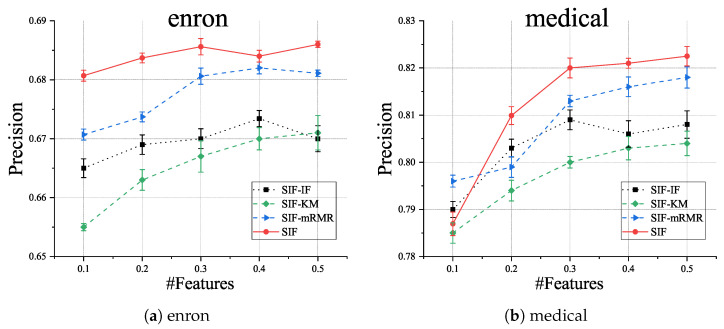
Comparisons of different inconsistency detection mechanisms.

As illustrated in Figure 8, SIF demonstrates a clear advantage over SIF-IF and SIF-KM, confirming that the inconsistency matrix Q is capable of detecting the specific instances whose characteristics are distinct from their local neighborhoods. Furthermore, the superiority of SIF over SIF-mRMR stems from the advantage of using CMI for feature complementarity. In general, a fine-grained representation facilitates a better description of specific instances, ultimately yielding the optimal feature subset of SIF that spans from global to local perspectives.

- **Effects of CMI-Based Strategy**

To validate the effectiveness of the CMI-based strategy, we compare SIF with an SIF-MLKNN, which is a variant of SIF using ML-KNN classifier to select instance-wise features. Specifically, each candidate feature is taken as an individual learner to construct the ML-KNN classifier. Then, we iteratively train these classifiers on the inconsistent instance set and select the top-$k_{2}$ features with the highest average precision performance as instance-wise features. In the experiment, $k_{1}/k_{2}=8/2$, and the inconsistency rate θ is fixed to 0.3.

**Figure 9:**
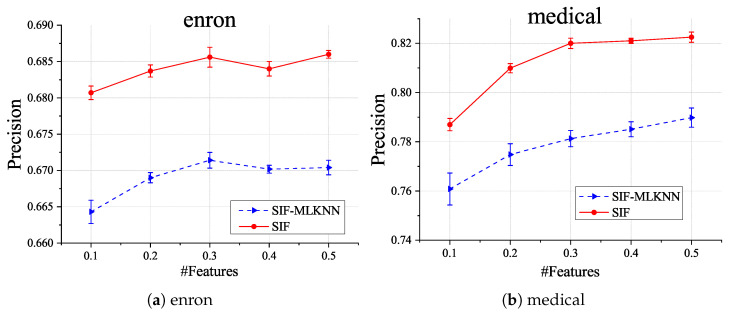
Comparisons of different inconsistency detection mechanisms.

As shown in Figure 9, SIF consistently outperforms SIF-MLKNN on the enron and medical datasets with varying the number of selected features. We further compare the two methods on additional datasets under various metrics in Table 5. The results indicate that SIF remains superior on the majority of datasets. In essence, SIF-MLKNN relies on the predictive power of individual features, while SIF explicitly accounts for feature complementarity. In many learning problems, feature interactions, such as feature redundancy or feature complementarity, play a critical role in selecting the optimal feature subset.

#### 4.3.3. Effects of Feature Selection Numbers

In order to evaluate the generalization ability of SIF, we select 40% and 60% of the original features for comparison. The F-measure performance of the ML-KNN classifier constructed with different numbers of selected features is shown in Table 6. It can be seen that the superiority of SIF is consistent across different scenarios. We can also observe that the performance of each approach is typically increased with selecting more features. Yet, this trend will not be maintained in most cases, such as the situation of the enron dataset in Figure 5. How to determine the optimal number of features to select is still an open question.

**Figure 10:**
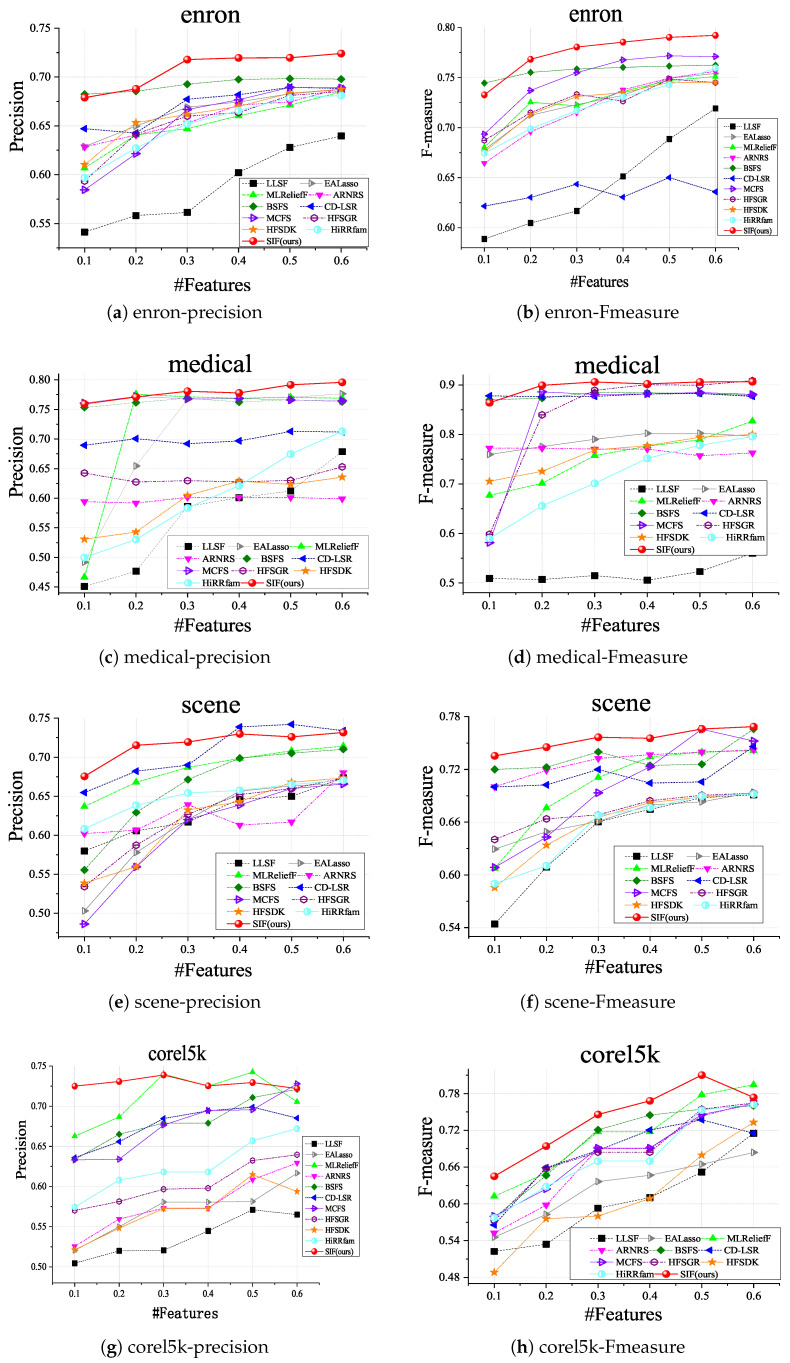
Feature selection performance on the LIFT classifier.

**Figure 11:**
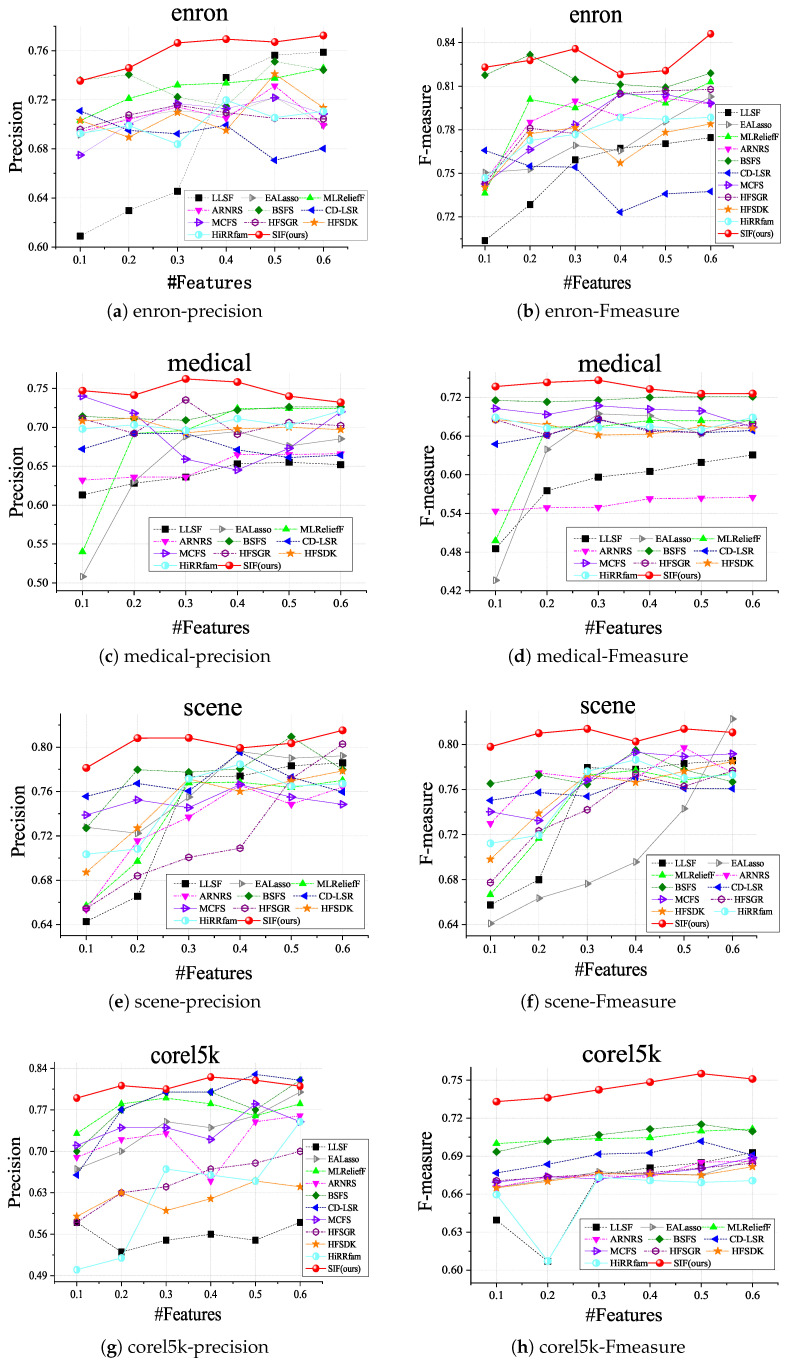
Feature selection performance on the BiLAS classifier.

We further leverage LIFT and BiLAS to assess the performance of the compared feature selection methods, as depicted in Figure 10 and Figure 11. LIFT and BiLAS are induced with distinct mechanisms on the basis of the SVM classifier and clustering analysis, respectively. Correspondingly, the performance of the compared feature selection approaches exhibits disparately on different classifiers. For example, the features selected by MLReliefF perform well on the enron dataset with BiLAS, while it degrades significantly with LIFT. Moreover, even for the same classifier, the performance of a feature selection method may vary substantially across datasets. For instance, CD-LSR performs poorly on the enron dataset with LIFT, yet it achieves competitive results on the scene dataset using the same classifier. As evidenced by the two figures, SIF demonstrates consistent robustness across different classifiers and datasets, underscoring its superior generalization capability in diverse learning scenarios.

#### 4.3.4. Effects of Inconsistent Instances

**Figure 12:**
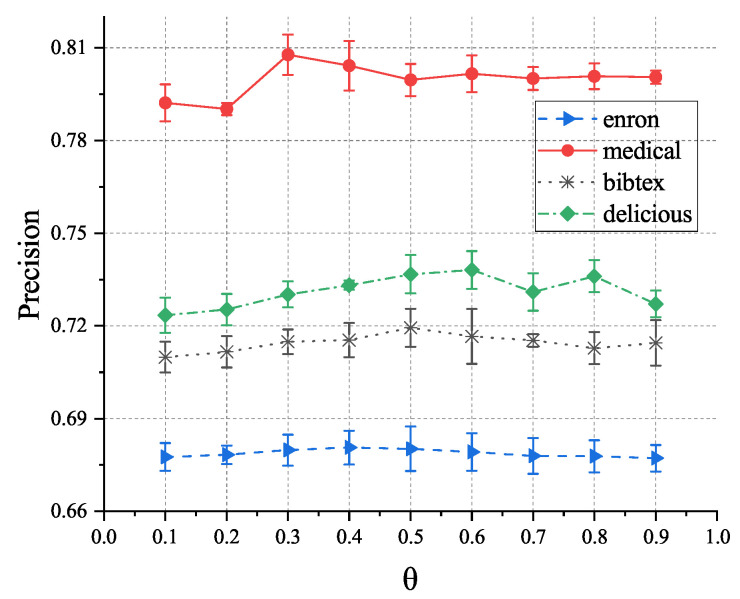
Effects of the number of inconsistent instances.

The number of inconsistent instances, which is controlled by θ, is crucial for SIF as it determines the scale of the instance-wise features. We conduct experiments on the four datasets with varying θ from 0.1 to 0.9 and record the precision performance in Figure 12. It can be seen that the optimal θ is distinctive across different datasets, that is, 0.3 for medical, 0.4 for enron, 0.5 for bibtex, and 0.6 for delicious.

Generally speaking, the performance of SIF is significantly improved when θ is increased from 0.1 to 0.3. This is because detailed discriminative information tends to be more explored when more inconsistent instances receive special attention. With continuously increasing θ, more instances are deemed as inconsistent ones, thereby inducing an excessive number of instance-wise features. In this situation, feature redundancy is proliferating, dramatically deteriorating the learning performance.

Following the analysis configuration of study [48], we conduct an analysis using different kernel functions, in order to evaluate the effects of the inconsistency matrix on identifying inconsistent instances. Specifically, we construct the instance affinity matrix Q via the widely-used kernels, including the linear kernel and polynomial kernel. Based on each affinity matrix I, the inconsistency matrix Q is recomputed and the corresponding inconsistency scores are re-estimated. Using the same threshold θ, instances are then divided into consistent and inconsistent groups. This evaluation aims to examine the effects of different kernel parameters on determining inconsistent instances.

**Figure 13:**
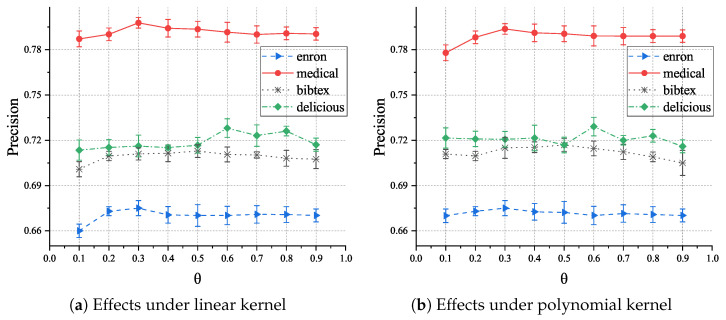
Effects of inconsistent instances obtained via different kernels.

As illustrated in Figure 13, the choice of kernel affects SIF’s selection decision, since the inconsistent instances flagged by matrix Q may vary, and those instances define which features are considered instance-wise. Nevertheless, the effect is modest, i.e., SIF’s performance makes slight variances when comparing Figure 12 and Figure 13. This implies that SIF’s inconsistency identification mechanism relies on the latent local structure of the dataset, which ultimately drives the selection of instance-wise features.

#### 4.3.5. Analysis of Inconsistency Matrix Q

- **Inconsistent Instance Distributions**

**Figure 14:**
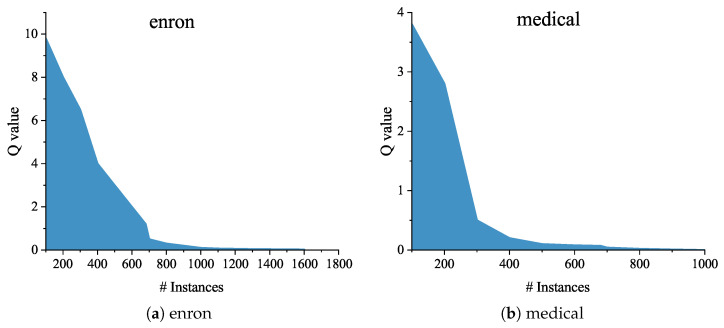
The distribution of inconsistent instances.

In order to intuitively reveal the distributions of inconsistent instances, we portray the distributions of inconsistency scores on enron and medical in Figure 14. For each instance **$x_{i}$**, its inconsistency score is computed as the $\ell_{2}$-norm of the corresponding row in Q, i.e., $\left|\right|Q^{i}{\left|\right|}_{2}$. Then, we rank all instances according to their Q values in descending order.

As observed in the figure, the inconsistency scores on both datasets exhibit a clear long-tail distribution. That is, a majority of instances are associated with relatively lower inconsistency scores, indicating that their local characteristics can be adequately explained by the learned semantic-aware representations. In contrast, a limited subset of instances possesses comparably higher inconsistency scores, suggesting substantial discrepancies between their local feature patterns and the global semantic commonality.

- **Comparative Analysis of Highly-inconsistent and Lowly-inconsistent Instances**

To further validate whether the inconsistency matrix **Q** can benefit the performance improvement of SIF by identifying inconsistent instances, we conduct a quantitative analysis comparing the instances with the higher and lower inconsistency scores. Similar to the configurations in analyzing inconsistent instance distributions, all instances are ranked according to their inconsistency scores **$\left|\right|Q^{i}{\left|\right|}_{2}$**. The top-ranked 20% instances are regarded as highly-inconsistent instances (Top-Q group), while the bottom-ranked 20% instances are regarded as lowly-inconsistent ones (Bottom-Q group). For both groups, we compute the classification error before and after incorporating instance-wise features, which are determined by the Top-Q group and Bottom-Q group, respectively. That is, the performance achieved completely by semantic-aware features is regarded as “Before”, and that achieved by 80% semantic-aware features and 20% instance-wise features is taken as “After”. The proportion is chosen due to the consideration that semantic-aware features provide the primary global semantic representations, while instance-wise features offer specific representations for a limited number of inconsistent instances.

**Figure 15:**
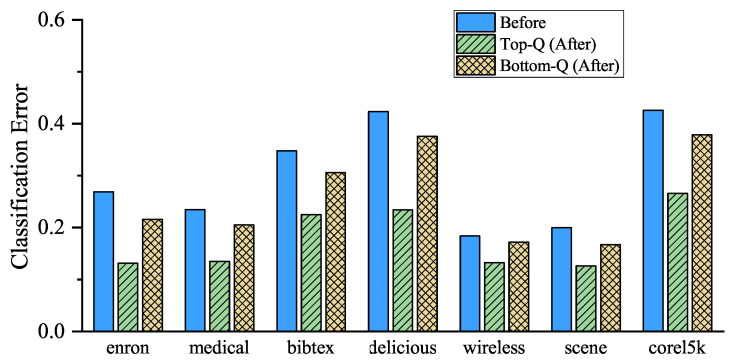
Classification performance comparisons of different kinds of inconsistent instances.

As illustrated in Figure 15, the performance of SIF can be significantly enhanced by incorporating the instance-wise features determined by highly inconsistent instances. It is worth noting that SIF can be slightly improved by incorporating the instance-wise features describing lowly-inconsistent instances. This implies that although fine-grained feature representations contribute less to characterizing lowly-inconsistent instances, their incorporation into the final optimal feature subset may provide a beneficial complement to coarse-grained semantic-aware features.

#### 4.3.6. Runtime Comparison

**Figure 16:**
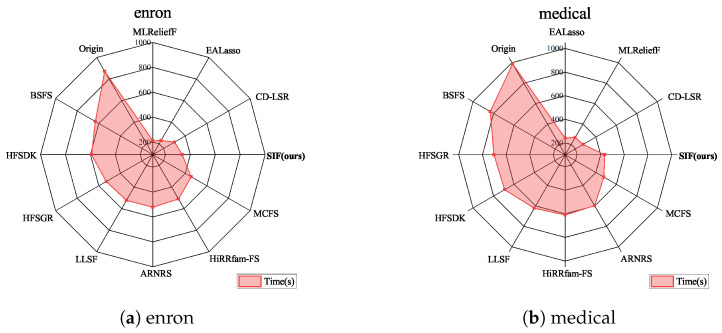
Runtime comparisons.

To evaluate the computational efficiency of the proposed framework, we further compare the runtime of SIF with competing feature selection methods. All experiments were conducted on the same hardware platform and software environment to ensure fairness. The reported runtime corresponds to the average execution time over ten independent runs. As shown in Figure 16, SIF requires more computational time than several traditional feature selection methods. This is mainly attributed to the construction of the semantic correlation matrix Q and the instance affinity matrix I. Nevertheless, the runtime of SIF remains comparable to that of other advanced semantic-aware and hierarchical feature selection approaches. More importantly, the additional computational cost is accompanied by consistent improvements in predictive performance across multiple evaluation metrics. Therefore, SIF achieves a favorable trade-off between effectiveness and computational efficiency.

#### 4.3.7. Statistical Analysis

**Figure 17:**
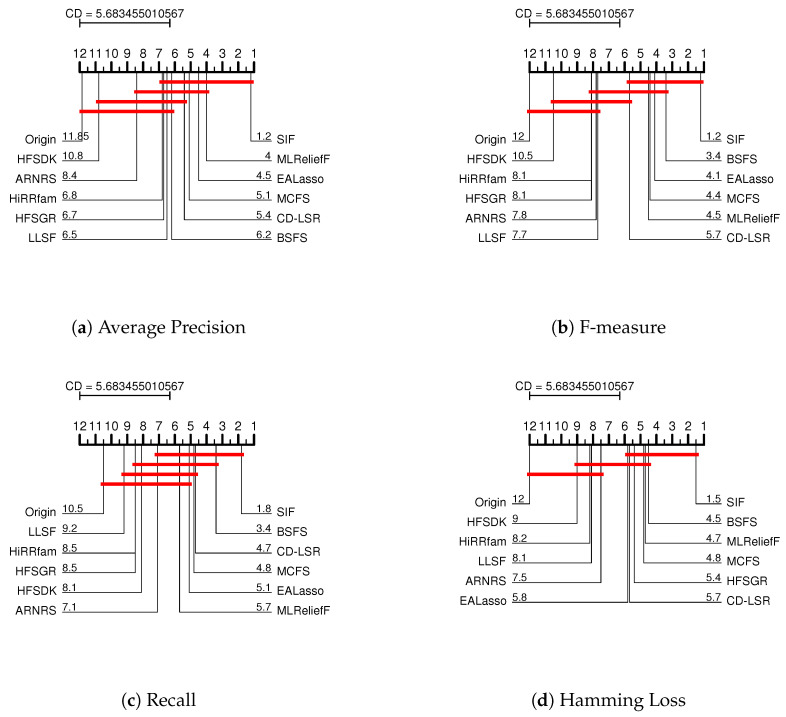
Statistical analysis.

We conduct statistical analysis to validate the significance of the performance achieved by SIF in the above experiments. We implement the Bonferroni–Dunn test across the compared approaches and display the CD (critical difference) diagram in Figure 17. The figure shows the average grade of methods under the four metrics, and a smaller grade (such as 1.25 achieved by SIF under precision) indicates reaching higher performance. The difference of two grade values is significant if their distance exceeds a red line range (i.e., a CD value). SIF beats the best across the four metrics, and this superiority is statistically significant in many cases, such as SIF versus HFSDK, ARMLNRS, and HiRRfam-FS under average precision.

#### 4.3.8. Case Study

**Figure 18:**
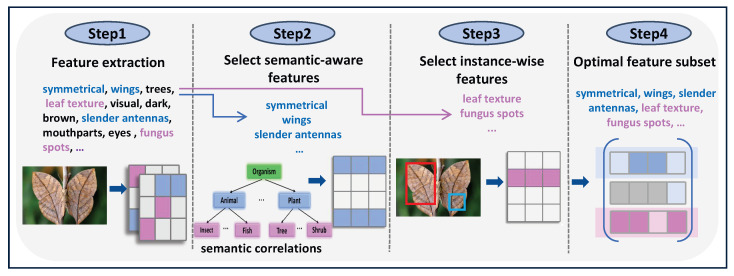
A visualization of an example of a complete task.

Figure 18 shows an intuitive example of SIF’s feature selection process. In this example, a Kallima inachus image is taken as the input and its visual features are extracted at the first step. Then, these features are fed to SIF for further selection. At the second step, semantic-aware features are selected based on their good capabilities of describing the semantic commonality, such as “symmetrical”, “wings”, and “slender antennas”. These features can help discriminate Kallima inachus at a coarse-grained level. At the third step, instance-wise feature selection is guided to find the instance-wise features, such as “leaf texture” and “fungus spots”. These features are specifying for Kallima inachus and facilitate the discrimination of Kallima inachus at a fine-grained level. Finally, the optimal feature subset is constructed by combining both kinds of features at the fourth step. This subset comprises diverse discriminative features, facilitating an integrated representation of the recognition target.

## 5. Discussions

This section highlights underemphasized issues and application considerations of SIF, expecting to guide future research and methodological improvements.

- **Potential Effects of the Scale of Inconsistent Instances**

In the instance-wise feature selection stage, features are selected merely for a small scale of inconsistent instances, which enables instance-level representation without incurring high computational overhead. However, this design may introduce a potential risk of overfitting when the number of inconsistent instances is substantially smaller than the number of features. For most benchmark datasets in Table 2, the number of instances is larger than the number of features. Consequently, even when the inconsistency rate θ ranges from 10% to 30%, the size of the inconsistent subset is not significantly smaller than that of features. Special cases are the enron and medical datasets, where the numbers of inconsistent instances are 170 and 98 when θ=10%, while the numbers of features are 1001 and 1449, respectively. According to the observations in Section 4.3.4, the overfitting phenomenon is not apparent, as the performance of SIF on these two datasets is not predominant, as demonstrated in Figure 12. While this issue is not significant on the tested datasets, we still give a cautious suggestion to avoid an overly small inconsistency rate, so as to mitigate any potential negative effects.

Since the threshold θ determines the proportion of instances identified as inconsistent, increasing θ may introduce additional instance-wise features and potentially increase the risk of overfitting. To quantitatively investigate this issue, we analyze the performance gap between the training set and the test set under different θ values on the enron dataset, where the performance gap is defined as

(9) $$\mathrm{Gap}=\frac{{\mathrm{Perf}}_{\mathrm{train}}-{\mathrm{Perf}}_{\mathrm{test}}}{{\mathrm{Perf}}_{\mathrm{train}}}\times 100\%,$$

where $Perf_{train}$ and $Perf_{test}$ represent the performance achieved on the training set and test set, respectively. As shown in Table 7, the training-test precision gap remains within a narrow range with different θ values. These results indicate that incorporating a larger number of inconsistent instances may not lead to noticeable overfitting. This stability can be attributed to the CMI-based feature selection strategy, which emphasizes feature complementarity and suppresses redundant feature inclusion. In other words, the proposed framework can be expected to provide good generalization ability across varying proportions of inconsistent instances.

- **Rational Choice of $k_{1}$, $k_{2}$, and θ**

$k_{1}$ and $k_{2}$ control how many features are selected from each type, and θ controls the proportion of inconsistent instances, which in turn governs the selection of instance-wise features. SIF determines the numbers of $k_{1}$, $k_{2}$, and θ via grid search in experiments. The effects of varying $k_{1}$ and $k_{2}$ values are evaluated in Section 4.3.2, revealing that SIF exhibits relatively stable performance on the benchmark datasets. Section 4.3.4 evaluates the effects of varying θ, indicating that restricting θ to a reasonable range enables SIF to gain performance benefits.

Although grid search systematically explores the parameter space, its high time cost makes it less applicable in the real world. How to adaptively determine the optimal number of features for different data in different scenarios for different real-world requirements remains an open question for the feature selection field, despite decades of effort on this topic. Similarly, adaptively determining appropriate $k_{1}$, $k_{2}$, and θ is complex in practical applications. We suggest that, given a fixed total number of selected features, it is preferable to increase the number of semantic-aware features (to a certain extent) in semantically consistent scenarios, such as the medical dataset in Figure 5, and to increase the number of instance-wise features in semantically diverse ones, such as the enron dataset. This challenging issue calls for further in-depth investigation in our future work.

- **Extension to Streaming and Dynamically Evolving Data**

The current SIF framework is designed under a batch-learning setting, where all data are assumed to be available prior to feature selection. Nevertheless, the core idea of SIF remains applicable to streaming scenarios. Specifically, the semantic correlation matrix can be incrementally updated by using newly observed semantic labels, and conditional mutual information can be estimated using online techniques to adaptively discover complementary features. In a dynamically evolving environment, new instances or labels may be added over time. Extending SIF to dynamically evolving datasets is not straightforward; however, with incremental updates of HSIC and approximate maintenance of instance neighborhoods, an online version of SIF is conceptually possible.

Although the current research mainly focuses on static data sets, extending SIF to streaming feature selection or for evolving data is a promising future research direction. Such extension can improve the scalability and adaptability of the framework in real environments.

- **High-Order Feature Interactions**

The proposed CMI-based feature selection strategy evaluates candidate features by conditioning on each semantic-aware feature individually. This formulation mainly captures pairwise conditional dependencies between candidate instance-wise features and semantic-aware features. In principle, higher-order feature interactions could be modeled by conditioning on multiple semantic-aware features simultaneously. However, such methods require estimating high-order joint probability distributions, which becomes increasingly difficult as the number of conditioning variables grows. As discussed in information-based feature selection studies [49], direct estimation of high-order mutual information terms is generally computationally intractable and statistically unreliable due to the curse of dimensionality.

Since high-order conditional mutual information estimation is extremely challenging in practice, we can resort to other non-information-theoretic methods for high-order feature dependencies. For instance, kernel-based conditional independence tests quantify such associations by projecting data into a high-dimensional feature space. Additionally, boosting-based methods can also capture high-order non-linear interactions through sequential model building.

## 6. Limitations

Despite the promising performance of SIF, several limitations should be acknowledged, calling for further investigation. This section outlines these limitations in detail.

- **Label Imbalance**

The issue of label imbalance may affect SIF’s final decision on instance-wise features. A major reason is that minority classes may tend to exhibit more semantic commonality rather than semantic diversity. Consequently, their instances have a lower probability of being identified as inconsistent and thus receive fewer instance-level feature representations. This drawback may be inherited by the downstream learning tasks, such as ML-KNN classification, potentially amplifying the negative effects of label imbalance. In the current SIF framework, the inconsistency detection mechanism based on the matrix Q does not explicitly consider label distribution balance, heralding the need for our future efforts on advanced improvements.

To investigate the label distribution characteristics of the identified inconsistent instances, we analyze the imbalance ratio of the inconsistent subset on the enron and medical datasets. Specifically, the labels whose frequencies exceed the average label frequency are regarded as major labels, while the remaining labels are treated as minor labels. The imbalance ratio is calculated as

(10) $$\mathrm{ImbalanceRatio}=\frac{n_{\mathrm{majority}}}{n_{\mathrm{minority}}},$$

where $n_{majority}$ and $n_{minority}$ denote the number of instances associated with major labels and minor labels respectively. As shown in Table 8, the inconsistent instance sets exhibit noticeably higher imbalance ratios than the original instance sets. This observation indicates that the instances associated with minority labels are more likely to be identified as inconsistent samples. This indicates that minority-label instances are less represented in the global semantic structure and therefore tend to receive larger inconsistency scores.

Similar imbalance-related challenges are commonly encountered in multi-label feature selection methods that rely on local instance modeling. For instance, the representative competing methods such as EALasso and MLRelief, do not explicitly consider label imbalance during feature evaluation. Although these methods exploit label relevance, semantic information, or feature-label dependencies, their feature selection criteria may still be dominated by majority-label patterns when the label distribution is highly skewed. Therefore, label imbalance remains a common challenge in multi-label feature selection, as it may bias semantic correlation estimation and subsequently affect feature importance evaluation. Addressing this issue would require a dedicated imbalance-aware semantic modeling framework, which is beyond the scope of the current study.

- **Noisy or Incomplete Labels**

The experiments in Section 4 were conducted under the assumption of no noisy or incomplete labels. However, such an ideal environment is rarely encountered in real-world applications. In terms of handling noisy semantic labels, SIF employs HSIC to estimate label correlations. With appropriately chosen kernel functions, HSIC is less sensitive to noisy data compared with information-based or distance-based metrics. This is one important reason why we resort to HSIC for explicit semantic correlation modeling. Solving the problem of noisy or incomplete labels is non-trivial and requires a systematic effort, which we consider an important direction for our future research.

Existing competing methods such as LLSF and SDIM rely heavily on semantic labels, label correlations, or graph structures constructed from label information. Consequently, when labels are noisy or partially missing, the learned semantic relationships may become unreliable and the resulting feature evaluation process may be adversely affected. Although these methods can exploit semantic dependencies among labels, they are not specifically designed to handle severe annotation noise or missing-label scenarios. Therefore, robustness to noisy or incomplete labels remains an open challenge in semantic-aware multi-label feature selection and deserves further investigation in future work.

- **Computational Complexity**

Another potential limitation of SIF is its high computational complexity, especially when scaling to large-scale datasets. As analyzed in Section 3.3, the total time complexity of SIF is approximately $O(l^{4}+d^{2}l^{2}+n^{2}l^{2}+dnl)$, where *d*, *l*, and *n* represent the numbers of features, semantic labels, and instances, respectively. The quartic term in *l* and the quadratic terms in *d* and *n* may make SIF computationally expensive for real-world applications. This overhead primarily arises from constructing the semantic correlation matrix S via HSIC computations and the instance affinity matrix I via pairwise Gaussian kernel evaluations. Compared with conventional feature selection methods, SIF incurs additional computational overhead due to the construction of the semantic correlation matrix and instance affinity matrix as well as CMI-based instance-wise feature selection. As illustrated in Figure 16, EALasso is the most computationally efficient method, requiring approximately 220 s on average. In contrast, SIF requires approximately 400 s, which reflects the additional cost introduced by inconsistency identification and instance-wise feature selection.

To improve SIF’s scalability, several directions can be explored in future work. For example, preliminary feature screening strategies may be employed to remove obviously irrelevant features before semantic-aware analysis. Approximate dependency estimation techniques could further reduce the computational cost of HSIC. In addition, parallel and distributed implementations may provide a practical solution for handling large-scale high-dimensional data.

- **Coupling of Two Stages**

We adopt two strategies to couple two stages as well as prevent feature redundancy (i.e., selecting duplicate or highly correlated features). First, instance-wise features are selected from the candidate subset, which is built by removing semantic-aware features from the original feature set. This strategy prevents selection overlap between the two stages. Second, we employ CMI to make the final decision on which features are chosen as instance-wise ones. By conditioning on the already selected features, CMI naturally avoids redundant feature selection and explicitly accounts for feature complementarity, thereby alleviating the decoupling of the two stages to some extent. Nevertheless, the two stages remain sequentially decoupled. To further enhance synergistic feature interaction, we plan to investigate a joint optimization framework that alternately updates both feature subsets, or a feedback mechanism that allows instance-wise selection to influence semantic-aware selection.

Beyond the feedback mechanism discussed above, a more effective interaction strategy may be achieved through iterative refinement between semantic-aware and instance-wise feature selection. Instead of performing a single feedback update, the two stages can be repeatedly executed until the selected feature subsets become stable. In each iteration, the updated semantic-aware feature subset can provide a refined semantic representation for inconsistency identification, while the newly selected instance-wise features can reveal local patterns that are insufficiently captured by the current semantic-aware features. Through such iterative interaction, global semantic information and local instance-specific information may gradually converge toward a more consistent and discriminative feature representation. This process can be viewed as a lightweight alternative to fully joint optimization while retaining the interpretability of the current framework.

A more integrated solution may be inspired by recent multimodal learning frameworks, such as LGVLM-mIoT [50], which employ bidirectional cross-attention to enable mutual interaction between heterogeneous information sources. In our framework, semantic-aware features and instance-wise features can be regarded as two complementary representations describing global semantic structure and local instance-specific characteristics, respectively. Instead of treating them as two independently selected feature subsets, a bidirectional cross-attention mechanism could be introduced to dynamically exchange information between them. Such bidirectional interaction would allow both feature subsets to be jointly adapted and continuously refined, thereby facilitating a more holistic representation than the current sequential framework.

## 7. Conclusions

This paper approaches semantic-aware feature selection specifying for instances. Correspondingly, SIF is proposed to tackle the dimension reduction issue, which can reduce model complexity and improve learning performance. The proposed SIF framework performs feature selection from both semantic and instance perspectives through a sequential two-stage process. Experimental results demonstrate that combining semantic-aware features with instance-wise features can effectively improve multi-label feature selection performance.

Although SIF provides a novel perspective of selecting multi-grained features at semantic and instance levels, it still faces some challenges. For example, SIF has some limitations when facing some unknown data sets. In addition, the current framework runs in a static learning environment and relies on a fixed set of inconsistent instances. We will focus on adaptive parameter learning, dynamic inconsistency modeling, more efficient optimization strategies, and the expansion of streaming data scenarios in our future work.

## Figures and Tables

**Table 2:** Data descriptions.

Datasets	Training	Testing	Features	Labels	Domain
enron	1362	340	1001	53	text
medical	782	196	1449	45	text
bibtex	5916	1479	1836	159	text
delicious	12,884	3221	500	983	web
wireless	7486	1871	13	10	network
scene	1607	800	294	6	image
corel5k	4000	1000	499	374	image

**Table 3:** Comparison evaluations under average precision and F-measure.

Methods	Datasets							*Average*
	**Enron**	**Medical**	**Bibtex**	**Delicious**	**Wireless**	**Scene**	**Corel5k**	
Average Precision (↑)								
Origin	0.4653	0.5362	0.5017	0.5624	0.5024	0.4121	0.4735	*0.4933*
LLSF	0.6503	0.6099	0.6995	0.6879	0.6564	0.5623	0.4872	*0.6219*
EALasso	0.6182	0.7016	**0.7275**	0.7295	0.6855	0.5922	0.5621	*0.6595*
MLReliefF	0.5998	0.7806	0.7131	0.6995	0.6950	0.6431	0.6627	*0.6848*
ARNRS	0.6032	0.6295	0.6467	0.6531	0.6273	0.5513	0.5334	*0.6063*
BSFS	0.6404	0.8030	0.6706	0.6282	0.5942	0.6407	0.6377	*0.6593*
CD-LSR	0.6522	0.7895	0.6399	0.6542	0.5350	0.6738	0.6375	*0.6531*
MCFS	0.5826	0.7899	0.6496	0.7105	0.6732	0.6033	0.6233	*0.6617*
HFSGR	0.6311	0.6408	0.6491	0.6494	0.6371	0.5883	0.5921	*0.6268*
HFSDK	0.4529	0.5996	0.6001	0.5948	0.5395	0.4932	0.5221	*0.5431*
HiRRfam	0.6209	0.6312	0.6381	0.6587	0.6324	0.5793	0.6421	*0.6289*
SIF (ours)	**0.7147**	**0.8259**	0.7247	**0.7566**	**0.7301**	**0.6821**	**0.7271**	***0.7373***
F-measure (↑)								
Origin	0.4422	0.5124	0.5429	0.5277	0.5533	0.4682	0.4387	*0.4979*
LLSF	0.5911	0.5706	0.6603	0.6562	0.6160	0.5402	0.5027	*0.5910*
EALasso	0.5873	0.6902	0.6901	0.7121	0.6647	0.5603	0.6201	*0.6464*
MLReliefF	0.5785	0.7272	0.6803	0.6821	0.6627	0.6224	0.5806	*0.6477*
ARNRS	0.5709	0.6512	0.6593	0.6591	0.6287	0.5504	0.4982	*0.6025*
BSFS	0.5963	0.7491	0.6719	0.6608	0.6231	0.6387	0.6777	*0.6596*
CD-LSR	0.5974	0.6730	0.6587	0.6210	0.6002	0.6247	0.6727	*0.6353*
MCFS	0.5967	0.7047	0.6331	0.7011	0.6529	0.6037	0.6260	*0.6454*
HFSGR	0.5605	0.6521	0.6419	0.6292	0.6129	0.5606	0.5421	*0.5999*
HFSDK	0.5152	0.5898	0.6305	0.5801	0.5604	0.5019	0.5021	*0.5542*
HiRRfam	0.5893	0.5957	0.6215	0.6332	0.6064	0.5521	0.6121	*0.6014*
SIF (ours)	**0.6468**	**0.7522**	**0.7119**	**0.7241**	**0.7026**	**0.6573**	**0.6982**	***0.6990***

**Note:** Bold values indicate the best performance and underlined values indicate the second-best performance.

**Table 4:** Comparison evaluations under the metrics of recall and Hamming loss.

Methods	Datasets							*Average*
	**Enron**	**Medical**	**Bibtex**	**Delicious**	**Wireless**	**Scene**	**Corel5k**	
Recall (↑)								
Origin	0.4213	0.4902	0.5915	0.4962	0.6160	0.5418	0.4084	*0.5093*
LLSF	0.5421	0.5360	0.6254	0.6267	0.5812	0.5214	0.5201	*0.5647*
EALasso	0.5506	0.6792	0.6561	0.6943	0.6455	0.5316	0.6915	*0.6341*
MLReliefF	0.5507	0.6799	0.6503	0.6649	0.6334	0.5866	0.4814	*0.6117*
ARNRS	0.5422	0.6727	0.6611	0.6652	0.6305	0.4545	0.5685	*0.5992*
BSFS	0.5584	0.5739	0.6725	0.6966	0.6546	**0.7094**	0.7111	*0.6537*
CD-LSR	0.5512	0.5869	**0.6791**	0.5915	0.6835	0.5846	0.7124	*0.6271*
MCFS	**0.6123**	0.6361	0.6174	0.6912	0.6332	0.6041	0.6287	*0.6318*
HFSGR	0.5117	0.6672	0.6153	0.6093	0.5911	0.5388	0.5002	*0.5762*
HFSDK	0.5951	0.5801	0.6636	0.5667	0.5825	0.5113	0.4835	*0.5689*
HiRRfam	0.5608	0.5626	0.6052	0.6091	0.5815	0.5277	0.5852	*0.5760*
SIF (ours)	0.6008	**0.6917**	0.6632	**0.6987**	**0.6951**	0.6578	**0.7421**	***0.6785***
Hamming Loss (↓)								
Origin	0.0485	0.0408	0.0821	0.0859	0.0559	0.0671	0.0893	*0.0675*
LLSF	0.0379	0.0231	0.0319	0.0322	0.0317	0.0521	0.0762	*0.0407*
EALasso	0.0338	0.0278	0.0315	0.0297	0.0305	0.0361	0.0467	*0.0337*
MLReliefF	0.0349	0.0198	0.0298	0.0287	0.0286	0.0389	0.0598	*0.0343*
ARNRS	0.0363	0.0223	0.0327	0.0311	0.0306	0.0445	0.0795	*0.0395*
BSFS	0.0419	0.0155	0.0314	0.0294	0.0368	0.0279	**0.0094**	*0.0274*
CD-LSR	**0.0301**	0.0155	0.0372	0.0386	0.0399	0.0256	0.0194	*0.0294*
MCFS	0.0457	0.0178	0.0351	0.0288	0.0299	0.0281	**0.0094**	*0.0278*
HFSGR	0.0319	0.0321	0.0306	0.0309	0.0316	0.0289	0.0305	*0.0309*
HFSDK	0.0417	0.0354	0.0373	0.0339	0.0378	0.0421	0.0235	*0.0359*
HiRRfam	0.0321	0.0343	0.0407	0.0368	0.0369	0.0343	0.0472	*0.0374*
SIF (ours)	0.0310	**0.0132**	**0.0253**	**0.0208**	**0.0241**	**0.0238**	0.0155	***0.0220***

**Note:** Bold values indicate the best performance and underlined values indicate the second-best performance.

**Table 5:** Comparison evaluations between SIF and SIF-MLKNN.

Methods	Datasets							*Average*
	**Enron**	**Medical**	**Bibtex**	**Delicious**	**Wireless**	**Scene**	**Corel5k**	
Average Precision (↑)								
SIF-MLKNN	0.6906	0.8092	0.7164	0.7362	0.7001	0.6701	0.7021	0.7178
SIF (ours)	0.7147	0.8259	0.7247	0.7566	0.7301	0.6821	0.7271	***0.7373***
F-measure (↑)								
SIF-MLKNN	0.6233	0.7405	0.6919	0.7094	0.6932	0.6617	0.7028	0.6889
SIF (ours)	0.6468	0.7522	0.7119	0.7241	0.7026	0.6573	0.6982	***0.6990***
Recall (↑)								
SIF-MLKNN	0.5837	0.6828	**0.6707**	0.6953	0.6863	0.6534	0.7244	0.6709
SIF (ours)	0.6008	0.6917	0.6632	0.6987	0.6951	0.6578	0.7421	***0.6785***
Hamming Loss (↓)								
SIF-MLKNN	0.0312	0.0134	0.0301	0.0219	0.0261	0.0241	0.0172	0.0234
SIF (ours)	0.0310	0.0132	0.0253	0.0208	0.0241	0.0238	0.0155	***0.0220***

**Table 6:** F-measure performance of the compared methods with selecting different number of features (↑).

Methods	Datasets							*Average*
	**Enron**	**Medical**	**Bibtex**	**Delicious**	**Wireless**	**Scene**	**Corel5k**	
40% features								
LLSF	0.5702	0.6521	0.6683	0.6631	0.6280	0.5801	0.5492	*0.6158*
EALasso	0.5922	0.7123	0.6852	0.7163	0.6569	0.6190	0.6203	*0.6574*
MLReliefF	0.5809	0.7386	0.6881	0.7018	0.6700	0.6182	0.5672	*0.6521*
ARNRS	0.5712	0.6910	0.6436	0.6594	0.6215	0.6215	0.5111	*0.6170*
BSFS	0.6171	**0.7532**	0.6745	0.6767	0.6159	0.6218	0.6802	*0.6627*
CD-LSR	0.5930	0.6969	0.6692	0.638	0.6281	0.6195	0.6885	*0.6476*
MCFS	0.6022	0.7115	0.6320	0.6943	0.6612	0.5014	0.6308	*0.6334*
HFSGR	0.5741	0.6623	0.6472	0.6396	0.6371	0.6167	0.5417	*0.6172*
HFSDK	0.5204	0.6076	0.6355	0.6302	0.5649	0.6216	0.5388	*0.5884*
HiRRfam	0.6191	0.6277	0.6390	0.6489	0.6382	0.6162	0.6294	*0.6312*
SIF (ours)	**0.6471**	0.7527	**0.7130**	**0.7416**	**0.7022**	**0.6302**	**0.7168**	***0.7005***
60% features								
LLSF	0.5823	0.6735	0.6748	0.6872	0.6671	0.5798	0.5520	*0.6309*
EALasso	0.6146	0.7254	0.6886	0.6918	0.6785	0.6262	0.6164	*0.6630*
MLReliefF	0.6083	0.7436	0.6856	0.6909	0.7012	0.6279	0.5647	*0.6603*
ARNRS	0.6253	0.7229	0.6803	0.6701	0.7283	0.6281	0.5112	*0.6523*
BSFS	0.6100	0.7515	0.6739	0.6765	0.7319	0.6308	0.6754	*0.6785*
CD-LSR	0.6068	0.7042	0.6597	0.6419	0.6447	0.6282	0.6941	*0.6542*
MCFS	0.6139	0.7234	0.6384	0.7008	0.6737	0.6298	0.6240	*0.6577*
HFSGR	0.5985	0.6745	0.6501	0.6682	0.6570	0.6277	0.5398	*0.6308*
HFSDK	0.5697	0.6462	0.6547	0.6312	0.6860	0.6271	0.5445	*0.6227*
HiRRfam	0.6335	0.6584	0.6595	0.6687	0.6554	0.6300	0.6327	*0.6483*
SIF (ours)	**0.6501**	**0.7736**	**0.7254**	**0.7431**	**0.7573**	**0.6561**	**0.7347**	***0.7200***

**Note:** Bold values indicate the best performance and underlined values indicate the second-best performance.

**Table 7:** Performance gap on enron.

θ	Train Precision	Text Precision	Gap
0.1	0.6770	0.6715	0.8124%
0.2	0.6737	0.6720	0.2523%
0.3	0.6798	0.6733	0.9561%
0.4	0.6806	0.6739	0.9844%
0.5	0.6802	0.6741	0.8967%
0.6	0.6789	0.6736	0.7806%
0.7	0.6779	0.6722	0.8408%
0.8	0.6761	0.6716	0.6655%
0.9	0.6684	0.6677	0.1047%

**Table 8:** Imbalance ratios of different datasets.

Datasets	Original Instance Set	Inconsistent Instance Set
enron	2.1	3.3
medical	1.8	2.9

## Data Availability

The original data presented in the study are openly available in an open-source Java library for learning from multi-label datasets https://mulan.sourceforge.net/(accessed on 12 July 2024).
